# Correlation Between Modified Functional Reach Test and Medio-Lateral Center of Pressure in Paraplegic Individuals With Motor-Complete Spinal Cord Injury

**DOI:** 10.3390/bioengineering11121185

**Published:** 2024-11-25

**Authors:** Valeska Gatica-Rojas, Juan Claudio López-Monardez, Ricardo Cartes-Velásquez

**Affiliations:** 1 Human Motor Control Laboratory, Department of Human Movement Sciences, Faculty of Health Sciences, Universidad de Talca, Av. Lircay S/N, Talca 3460000, Chile; 2 Universidad Autónoma de Chile, Chile; juanclaudiolm@gmail.com; 3 School of Medicine, Universidad de Concepción, Concepción 4030000, Chile; cartesvelasquez@gmail.com

**Keywords:** functional reach test postural control, balance, sitting balance, spinal cord injury

## Abstract

The Modified Functional Reach Test (mFRT) was developed to assess sitting balance in individuals with spinal cord injury (SCI). No studies have explored which mFRT reach directions correlate with the center of pressure (CoP) variables in patients with motor-complete SCI (mcSCI). Addressing this gap is important for improving the clinical usefulness of the mFRT. Thus, this study aims to determine the correlation between seated balance parameters based on CoP and the mFRT in individuals with MCSCI. A total of 10 individuals with mcSCI (9 males and 1 female; range 20–42 years; 4 high paraplegia and 6 low paraplegia). Individuals were tested using a force platform during the sitting postural balance test (SPBT) and the mFRT with/without force plate with three measures of reach: forward (FR), right (RR), and left (LR). The sway parameters investigated were the area CoP sway (CoP_Sway_), the average velocity of CoP displacements along the anterior–posterior (V_AP_) and medial-lateral (V_ML_) directions, and standard deviation in both directions (SD_AP_ and SD_ML_). The Pearson correlation test was used to analyze the data. Significant correlations were found between the mediolateral reaches of the mFRT and corresponding CoP variables. The rightward and leftward reaches of the mFRT both showed strong correlations with CoP variables during the leftward reach. Additionally, the leftward mFRT exhibited moderate correlations with CoP variables in the same, rightward, and forward directions. The mFRT medio-lateral direction correlates with CoP at medio-lateral directions in paraplegic individuals with mcSCI.

## 1. Introduction

Spinal cord injury (SCI) is a prevalent neurological condition globally, leading to various motor and sensory impairments. The causes of SCI include traumatic events, such as vertebral fractures, as well as non-traumatic factors, like infections and vascular damage [1]. Ding et al. [2], through a systematic review, reported 0.9 million incident cases, 20.6 million prevalent cases, and 6.2 million years lived with disability due to SCI globally in 2019. While SCI affects individuals across a broad age range, it is predominantly concentrated among young adults. As with other neurological conditions, SCI frequently results in compromised balance function [3,4,5,6].

Individuals with SCI who have impaired postural control face significant challenges in performing daily activities, which increases their risk of falls and subsequent musculoskeletal injuries, thereby diminishing their quality of life [1,7]. Impaired balance control in a sitting position is common in both paraplegic and tetraplegic patients and is associated with reduced motor performance during daily movements. This impairment also leads to compensatory patterns of muscle activation involving muscles not typically used for postural support [8].

Given the critical impact of balance impairments, improving postural control is a primary focus of treatment for clinicians and rehabilitators working with SCI patients [9]. The gold standard for measuring postural balance is posturography using a force plate [10], an instrument commonly found in human movement laboratories but less so in hospitals and rehabilitation centers. Furthermore, this equipment requires specialized training to operate and analyze the center of pressure (CoP) variables, which are essential for constructing postural control profiles. The complexity of interpreting posturographic data presents challenges in non-academic clinical environments, particularly within SCI rehabilitation programs.

A more straightforward method for quantifying seated postural balance in clinical settings is necessary. The Modified Functional Reach Test (mFRT) was developed to assess sitting balance in individuals with SCI. The reliability and validity of the mFRT have been established in various populations [11,12,13]. The mFRT is quick, easy to administer, and provides results that are straightforward for physiotherapists and rehabilitation centers to interpret [12,14]. Moreover, current research in patients with SCI still uses mFRT to evaluate the effects of several interventions, such as virtual reality [15,16,17], exoskeletal devices [18], and electrical stimulation [19,20]. The continued application of the mFRT in emerging rehabilitation technologies underscores its versatility and potential as a standardized tool for tracking both functional recovery and the efficacy of innovative therapies in SCI rehabilitation. However, to our knowledge, no studies have examined which direction(s) of reach in the mFRT are associated with CoP variables in patients with motor-complete SCI (mcSCI). This gap in the literature is crucial as it could enhance the clinical utility of the mFRT for practitioners.

Therefore, this study aims to determine the correlation between seated balance function parameters based on CoP and the mFRT in individuals with mcSCI.

## 2. Materials and Methods

### 2.1. Design, Participants, and Setting

A cross-sectional study was conducted with 10 volunteers (9 males and 1 female) aged 20 to 42 years. Inclusion criteria included participants aged 18 to 50 years, with an ASIA Impairment Scale grade A (ASIA) motor-complete spinal cord injury (mcSCI) of at least 6 months duration and the ability to maintain a sitting posture for at least 30 s. Exclusion criteria were the presence of associated disabilities diagnosed by a neurologist, such as uncorrected visual or vestibular impairments, and the presence of ulcers that could alter sitting posture.

All measurements were conducted in the Human Motor Control Laboratory at Universidad de Talca during the afternoon hours (16:00–20:00). To ensure a controlled environment, all tests were performed in a quiet, closed room with natural light from windows, maintained at a temperature of 25 °C. The study was approved by the Bioethics Committee of Universidad de Talca (Ref. No. 105-VG), and written informed consent was obtained from all participants.

### 2.2. Variables

Demographic and clinical characteristics of the participants, including age, sex, weight, height, body mass index, duration of spinal cord injury, functional level, and dominant reach, were recorded.

**Figure 1:**
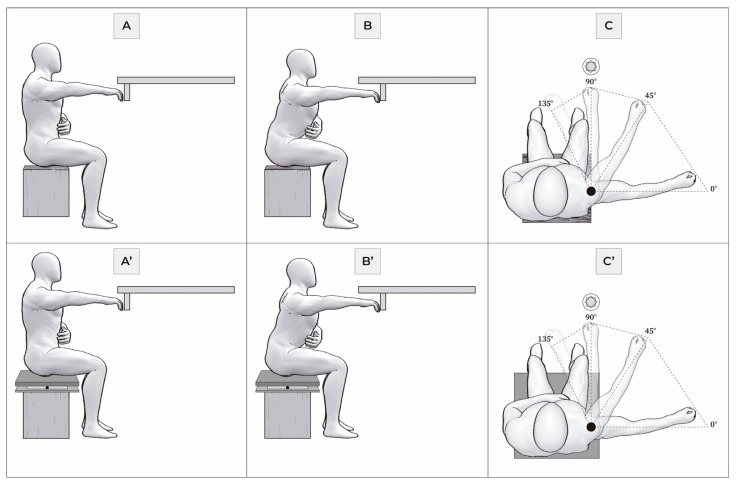
In (**A**–**C**), the Modified Functional Reach Test (mFRT) in a seated position in three directions. (**A’**–**C’**) the mFRT clinical test on force plate sitting.

The mFRT is a clinical assessment adapted for individuals with SCI, with established reliability and validity [14]. It measures the maximum functional reach of the upper limb from a seated position in three directions: forward reach (FR), right reach (RR), and left reach (LR), ensuring safety and stability [21]. Participants were seated with their backs unsupported on a stable wooden surface, with legs separated at shoulder width and feet resting on the floor, maintaining a 90° angle at the hips, knees, and ankles. The dominant arm was identified for each participant, positioned in 90° flexion, while the non-dominant arm was either supported on the thighs (for those with cervical lesions) or crossed over the chest (for those with thoracic injuries). From this initial position, participants performed maximum FR, RR, and LR for 3 s, returning to the initial stable position afterward. Each participant completed two practice trials, followed by three test trials in each direction, with the average reach distance (in centimeters) recorded. The ulnar styloid process of the dominant arm served as the reference point (Figure 1A–C). Complete recovery pauses were allowed between trials. The mFRT was completed within 5 min.

### 2.3. Posturographic Measurements

The mFRT was conducted on a force plate centered on a wooden block (46 cm W × 43 cm L × 31 cm H), ensuring that hip, knee, and ankle angles were approximately 90°, with feet resting on the floor. The non-dominant arm was maintained as previously described. For each participant, the dominant reach direction identified in the mFRT was used. While seated with their backs unsupported, participants performed three trials in each mFRT direction (FR, RR, and LR), and the average reach distance (in centimeters) was recorded. The mFRT was measured using the center of pressure (CoP) with eyes open, maintaining each direction for a maximum of 30 s (Figure 1A’–C’). Data were collected at a sampling rate of 200 Hz using an AMTI OR6–7 force plate and AMTI-NetForce software (AMTI Inc., Boston, MA, USA). A custom Matlab R2012 (MathWorks Inc., Natick, MA, USA) script was used to apply a second-order Butterworth low-pass filter (cut-off frequency 40 Hz) and calculate CoP variables for each mFRT direction (FR, RR, and LR), including CoP sway area (CoP_Sway_), standard deviation (SD_ML_ and SD_AP_) and velocity (V_ML_ and V_AP_) of CoP in the mediolateral (ML) and anterior–posterior (AP) directions. Standard deviation measures (SD_ML_ and SD_AP_) reflect the variability of CoP displacements, indicative of motor responses aimed at minimizing postural sway. The testing was completed within 10 min. To ensure participant safety, two staff members were always present at the participant’s side during each test.

### 2.4. Data Analysis

Statistical analyses were performed using IBM-SPSS 20.00 (SPSS Inc., Armonk, NY, USA). The assumptions of normality and homogeneity of outcome measures were assessed using the Shapiro–Wilk and Levene tests, respectively. Pearson correlation analyses were conducted to determine the relationship between CoP variables and mFRT in seated postural balance function. For all analyses, the significance level was set at *p* < 0.05.

## 3. Results

The characteristics of the participants are summarized in Table 1. The Pearson correlation coefficients between the distance reached in the mFRT (clinical test alone) and the CoP variables measured during the three reach directions of the mFRT are presented in Table 2.

Significant correlations were observed between the mediolateral reaches of the mFRT (rightward and leftward) and the corresponding CoP variables. Specifically, the rightward reach of the mFRT showed a statistically significant (*p* < 0.01) and strong correlation with CoP variables measured during the leftward reach (CoP_Sway__LR, SD_ML__LR, and V_ML__LR). Similarly, the leftward reach of the mFRT demonstrated a significant (*p* < 0.01) and strong correlation with CoP variables measured during the leftward reach (CoP_Sway__LR and SD_ML__LR).

In addition, the leftward mFRT showed a moderate but significant correlation (*p* < 0.05) with the CoP variables measured during the clinical test in the same direction (V_ML_), as well as with variables during the rightward reach (CoP_Sway_, SD_ML_, and V_ML_) and forward reach (CoP_Sway_). These findings are detailed in Table 2.

## 4. Discussion

Postural balance is a complex motor skill that requires the integration of sensory and motor systems. Effective balance control is essential for performing daily activities such as sitting, standing, and walking. In individuals with spinal cord injury (SCI), impaired balance control predisposes them to various complications. The mFRT is a widely used tool for assessing sitting balance, and its reliability has been well-documented in various neurological conditions. However, there is limited literature on the reliability of the mFRT specifically in paraplegic individuals with SCI to level thoracic (upper and low).

This study identified significant correlations between the mediolateral reaches of the mFRT and corresponding CoP variables. Both rightward and leftward reaches of the mFRT demonstrated strong correlations with CoP variables during the leftward reach, while the leftward mFRT also showed moderate correlations with CoP variables in the same, rightward, and forward directions.

Previous research by Lynch et al. [14] demonstrated high test–retest reliability of the mFRT in individuals with mcSCI across different levels of lesion: C5–6 tetraplegia, upper thoracic T1–4 paraplegia, and low thoracic T10–12 paraplegia, with intraclass correlation coefficients (ICC) ranging from 0.84 to 0.95. Additionally, they found that the mFRT could effectively measure differences among these levels of SCI, with no significant differences between right and left upper extremity reaches. A systematic review by Arsh et al. [1] further supported the reliability of the mFRT, reporting good to excellent reliability across six studies, with ICC values ranging from 0.78 to 0.99.

Interestingly, most studies focused on the forward reach, with only two assessing reaches in multiple directions (forward, left, and right). Therefore, this is the first study to show a strong correlation between the mediolateral reaches of the mFRT and correlation with CoP variables, such as CoP_Sway_, SD_ML_, and V_ML_, during the leftward reach in paraplegic people with mcSCI. In line with this, highlighting the importance of evaluating functional scope in various directions [22] and separately according to the level of the lesion (paraplegics and quadriplegics) in people with mcSCI.

In contrast to previous studies [2], our research did not find significant correlations between CoP variables and forward reach in individuals with mcSCI. This discrepancy suggests that the mediolateral (side-to-side) dynamics may offer unique insights into balance control in this population, which are not captured by solely evaluating forward reach. Evaluating mediolateral directions could be crucial for understanding the compensatory mechanisms that these individuals develop, given their altered motor control and balance strategies. However, it is important to recognize the limitations of this study, particularly the relatively small sample size. A limited number of participants may reduce the statistical power, thereby influencing the generalizability of the findings. Therefore, while these results offer valuable perspectives on balance control in paraplegic individuals, they should be interpreted with caution. Further research with larger and more diverse samples is needed to determine whether these findings are consistent across broader populations with mcSCI and to fully elucidate the implications of mediolateral balance strategies in this group.

## 5. Conclusions

The mFRT medio-lateral direction correlates with CoP at medio-lateral directions in paraplegic individuals with mcSCI.

## Figures and Tables

**Table 1:** Demographic and clinical characteristics of the participant.

Variables	Mean ± SD	Range
Age (Years)	29 ± 7.77	20–42
Height (cms)	173 ± 8.98	157–186
Weight (kg)	77.6 ± 20.72	53–126
BMI (kg/m^2^)	25.6 ± 5.79	19.2–36.4
Time post-lesion (months)	56.6 ± 50.56	6–144
Sex	(Female/Male)	1/9
Level functional of injury	(UT/LT)	4/6
ASIA. Grade A		10

UT: Upper thoracic; LT: Low thoracic; ASIA: American Spinal Cord Injury Association; Grade A: The impairment is complete. There is no motor or sensory function left below the level of injury.

**Table 2:** Correlation of the variables of the COP and the variables of the mFRT for the total study group.

Measures	Forward Reach	*p* Value	Right Reach	*p* Value	Left Reach	*p* Value
FR CoP_Sway_	0.30	0.340	0.45	0.141	0.64 *	0.025
SD_ML_	0.28	0.382	0.36	0.252	0.42	0.172
SD_AP_	0.18	0.566	0.37	0.241	0.6 *	0.041
V_ML_	0.08	0.816	−0.08	0.804	0.27	0.400
V_AP_	0.17	0.589	0.27	0.391	0.49	0.102
RR CoP_Sway_	0.43	0.166	0.39	0.215	0.64 *	0.030
SD_ML_	0.36	0.246	0.49	0.102	0.64 *	0.025
SD_AP_	0.50	0.095	0.58 *	0.046	0.64 *	0.026
V_ML_	0.39	0.204	0.58 *	0.050	0.69 *	0.013
V_AP_	0	1.000	0.20	0.528	0.31	0.331
LR CoP_Sway_	0.25	0.428	0.82 *	0.001	0.78 *	0.003
SD_ML_	0.12	0.719	0.74 *	0.006	0.79 *	0.002
SD_AP_	0.45	0.138	0.64 *	0.024	0.6 *	0.041
V_ML_	0.44	0.152	0.71 *	0.01	0.66 *	0.020
V_AP_	0.31	0.326	0.72 *	0.010	0.63 *	0.030

FR: forward reach; RR: right reach; LR: left reach. CoP: center-of-pressure; CoP_Sway_: area of CoP sway in cm^2^; SD_ML_ and SD_AP_: standard deviation of CoP in the directions mediolateral and anterior–posterior, both in mm; V_ML_ and V_AP_: CoP velocity in the directions mediolateral and anterior–posterior. * Significant correlations *p* < 0.05.

## Data Availability

The data presented in this study are available on request from the corresponding author due to privacy issues.
